# Ankle Bracelet Laser as a Novel Portable Device to Improve Walking in Patients With Parkinsonism: Randomized Crossover Controlled Trial

**DOI:** 10.2196/70209

**Published:** 2025-10-07

**Authors:** Peeraya Ruthiraphong, Kwan Srisilpa, Chompoonuch Ratanasutiranont

**Affiliations:** 1 Department of Rehabilitation Medicine Faculty of Medicine Ramathibodi Hospital Mahidol University Bangkok Thailand

**Keywords:** parkinsonism, gait disorders, cues, self-help devices, lasers

## Abstract

**Background:**

Freezing of gait (FOG) is a common and debilitating symptom of parkinsonism. Although visual cues have proven efficacy in alleviating FOG, most current visual cues are fixed in place, restricting their use to controlled environments such as clinics or homes. Mobile open-loop cueing devices have been developed to address this limitation; however, they typically require manual activation to deliver the visual cues, which can be particularly challenging for patients with attention or cognitive impairments, leading to equivocal results in improving gait performance.

**Objective:**

The aim of the study is to assess the efficacy of an ankle bracelet laser, a new mobile visual cue designed for practical use, in improving gait performance in patients with parkinsonism and FOG.

**Methods:**

A randomized controlled 2-period crossover trial was conducted from June 15, 2020, to October 1, 2020, at Ramathibodi Hospital. In total, 10 patients with parkinsonism and FOG were enrolled in 2 conditions: walking with laser-off first and walking with laser-on first. Gait speed, the timed up and go test, stride length, and the locomotor rehabilitation index were assessed twice in each trial with a 10-minute washout period.

**Results:**

The results showed favorable improvement in all parameters. Gait speed and stride length improved by 0.07 m/s (95% CI 0.04-0.09 m/s; *P*<.001) and 0.17 m (95% CI 0.11-0.23 m; *P*<.001), respectively, with laser-on. The timed up and go test duration was reduced by 7.69 seconds (95% CI 2.82-12.55 seconds; *P*=.002). The locomotor rehabilitation index improved by 4.46% (95% CI 2.56%-6.36%; *P*<.001). When using the device, there were no adverse effects, such as dizziness or blurred vision.

**Conclusions:**

The ankle bracelet laser cue produced immediate improvements in gait speed, stride length, and balance in patients with parkinsonism and FOG, suggesting that the device can acutely enhance gait performance. Further research is needed to determine whether these benefits are sustained and applicable to daily life activities.

**Trial Registration:**

Thai Clinical Trials Registry TCTR20210511001; https://www.thaiclinicaltrials.org/show/TCTR20210511001

## Introduction

### Background

Individuals with parkinsonism walk with a slower gait speed, shorter stride length, and higher cadence, resulting in an asymmetrical gait and increased energy expenditure compared to healthy people [1,2]. Freezing of gait (FOG) is one of the most common debilitating symptoms of parkinsonism and leads to multiple falls, related injuries, mobility limitations, functional dependence, restrictions in social activities, as well as psychological effects such as anxiety and depression.

Pharmacological and surgical interventions do not always provide satisfying outcomes [3-5]. External cueing has been used as a nonpharmacological intervention to alleviate FOG and improve gait parameters [6-8]. Visual cues may have more advantages in improving spatial parameters than auditory cues [9]. Fixed visual cues, such as taping a line on the floor to step over, have shown good immediate outcomes. However, this method is limited because it can only be used in environments where a fixed visual cueing line has been placed.

Currently, mobile visual cues mostly take the form of open-loop cueing (constant stimulus) [10] systems, such as laser light incorporated with a gait aid such as a walker or cane. Several studies have reported favorable outcomes regarding the immediate effect of reducing FOG and improving gait parameters; however, some results were equivocal [11], and some studies did not demonstrate improved outcomes [12]. In addition, gait speed as a predictor of ambulation ability [13] did not always improve with this method [14] and even slowed with the use of the device [15]. This may be because the open-loop system uses manual control to project the laser light and requires attention to project the cues at a constant distance in front of the foot. This dual-task process may be difficult for some patients to perform, especially for patients with some attention or cognitive impairment. Because cognitive impairment is commonly related to FOG in parkinsonism, this could be one of the factors leading to negative outcomes [16,17]. In addition, patients with parkinsonism and FOG showed greater sequence learning deficits than nonfreezers [18], and these deficits worsened further under dual-task conditions.

The long-term effect of visual cueing devices remains unclear. Some studies showed that these devices could not provide carry-on or retention effects [15], resulting in cue dependency for patients with FOG. This may be because of cognitive overload when using the device, which could distract attention away from walking [8,16], reducing the neural reserve for the motor relearning process [17].

Physical exercise could improve the motor function in parkinsonism. Nordic walking (NW) [2] is one of the exercise techniques using a specialized pole, working with arm swing to coordinate the rhythmic movement of the lower extremities and enhance the body’s ability to move forward with an increased range of motion in the upper and lower extremities. NW training could improve gait performance and reduce energy expenditures in patients with Parkinson disease (PD). However, studies of the efficacy of NW were limited to patients with mild to moderate PD, Hoehn and Yahr (H&Y) stage 1-1.5 [2], or H&Y stages 1-3 [19], and only 1 person experienced FOG. Another trial [20] also showed improvement of gait speed, timed up and go (TUG), and the locomotor rehabilitation index (LRI) in patients with PD with an average of H&Y stage 1.5, but only 3 of 16 participants had FOG. Additional research has shown mixed results. One study reported that following an individualized and progressive NW training intervention, significant improvements were observed in walking function, daily activity level, and motor function, with participants sustaining independent NW exercise over 3 months and showing increased serum brain-derived neurotrophic factor levels after 5 months [21] Conversely, a systematic review and meta-analysis [22] found that NW did not lead to clinically significant changes in global motor impairment, functional mobility, balance, or physical fitness in patients with PD. However, it suggested that NW may improve walking ability and quality of life, though further research is needed to confirm these outcomes.

Recently, we developed ankle bracelet lasers as a new visual cueing device [23] aimed for ease and practical use to reduce cognitive overload. The ankle bracelet laser was designed as a closed-loop (intermittent stimulus) cueing system that provides a laser cue automatically following individual walking steps, so each patient can walk naturally. However, the device cost is too expensive for most people in our country due to the motion sensor and the software program that detects the gait motion to command the laser cue.

Therefore, we created another new version using ultrasonic sensors to detect the motion during the stance and swing phases. The command is simpler and fits with the low-income insurance cost. This device is currently a prototype. Electrical safety testing has been conducted by our engineering team to ensure safe operation; however, the device has not yet received formal medical device certification. The current version is planned to be sold at approximately US $185, reduced from US $490 in our earlier design.

The ankle bracelet laser operates intermittently, activating during the stance phase when the foot touches the floor, as detected by the integrated sensor. Once the foot contacts the floor, the laser line automatically projects in front of the patient’s contralateral foot, providing a target to step to or over. This cycle of laser line projections repeats alternately and continuously throughout the walking period without the need for manual control or additional commands. The use of a mobile, laser-guided system might also promote neuroplasticity and motor relearning. This is achieved through a sensory-motor cycle in which an individual’s own movement triggers intermittent sensory cues. This repeated feedback loop reinforces ongoing motor performance and may lead to long-term training effects [10,11]. The Velcro strap at the ankle position is easy to wear for a patient with a stiff back or tremors, which are commonly found in parkinsonism, because they do not need good hand function or to bend their back to put on the device. In addition, this device can be used with bare feet, which is suitable for Asian cultures, or with any kind of shoes.

### Objective

The aim of the study is to study the efficacy of the ankle bracelet laser in improving walking performance in parkinsonism.

## Methods

### Study Design

We conducted a randomized crossover design study to lessen the between-patient variation. The study was conducted between June 15, 2020, and October 1, 2020. Patients with parkinsonism and FOG were enrolled in 2 conditions: walking with laser-off first and walking with laser-on first. The washout period was set at 10 minutes between the 2 conditions to avoid carryover effects.

### Participants

Patients with parkinsonism were recruited from the Physical Medicine and Rehabilitation Outpatient Clinic of Ramathibodi Hospital. The inclusion criteria were as follows: (1) patients with parkinsonism diagnosed by a neurologist, (2) aged 18-90 years, (3) ability to walk at least 16 m with or without a gait aid, (4) no medication adjustment within the previous 3 months, and (5) H&Y stages 1-4. The exclusion criteria were as follows: (1) unable to see the laser line projection on the floor, (2) other neurological disorders, (3) unable to communicate well, (4) unable to use the device, and (5) denied or rejected from the study.

### Ethical Considerations

This study was conducted in accordance with the principles of the Declaration of Helsinki. The protocol was reviewed and approved by the Human Research Ethics Committee, Faculty of Medicine, Ramathibodi Hospital, Mahidol University (approval COA. MURA2020/702). Written informed consent was obtained from all participants prior to enrollment, including consent for data collection and publication of deidentified results. To ensure confidentiality, all data were anonymized before analysis, and no personal identifiers were collected or stored in the dataset. Participants received compensation for their time as well as reimbursement for transportation costs related to study visits. This study was retrospectively registered with the Thai Clinical Trials Registry (TCTR20210511001) [24].

### Intervention

**Figure 1:**
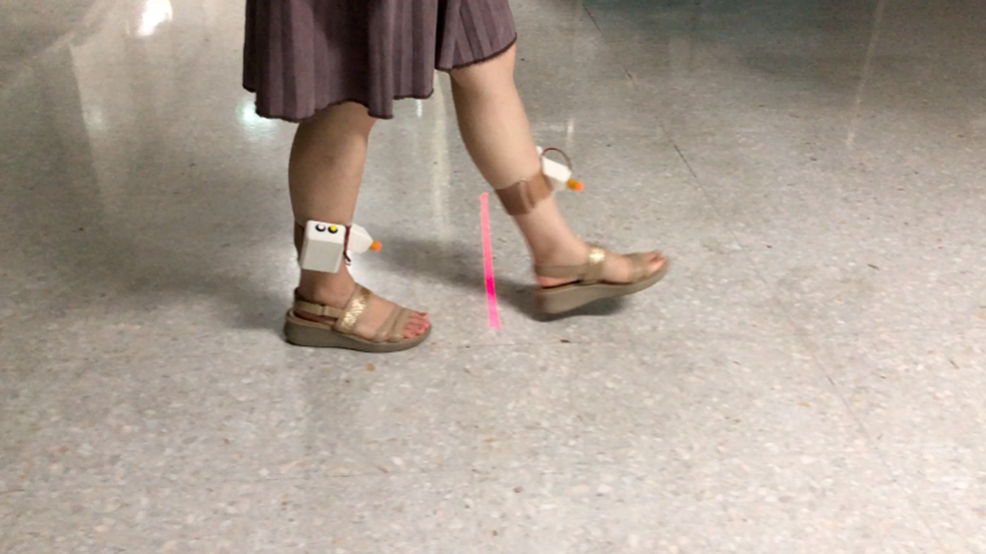
Ankle bracelet laser attached to both tibias.

The ankle bracelet laser was designed as a closed-loop cueing system that provides a laser cue automatically following an individual’s walking steps. The ankle bracelet lasers were attached to both tibial areas. The device size was 4×5×2 cm, weighed 200 g, and is shown in Figure 1. The device uses an ultrasonic sensor to detect foot position from the ground and is integrated with a microcontroller to control the laser line projection correlated with an individual’s walking steps. Once a patient performed a heel or foot strike, the laser line was projected in front of the contralateral foot to step on or over when entering the swing phase. The laser line disappeared once the foot was lifted off the ground. These cycles repeat alternately and automatically, following each individual’s walking step.

### Experimental Design

The participants were informed of the objective and procedure of the study and provided signed informed consent before starting the trials. Each participant was randomized using a sealed envelope into 1 of 2 conditions: walking with the laser-off first or walking with the laser-on first. There was a 10-minute washout period between each condition.

Each participant walked a 10-m path twice at a comfortable speed, taking a break for a few minutes or until they felt ready to perform the TUG test twice. Gait speed and related spatiotemporal parameters were analyzed using a 6-m segment located in the middle of the total 10-m walking distance, excluding the initial and final 2 m. The start and end points of the 6-m segment were marked with striped floor lines to clearly identify the first and last strides.

A digital single-lens reflex camera, the Canon EOS 500D equipped with a Canon EF-S 18-55mm f/3.5-5.6 IS II kit lens, was used to record videos at 60 frames per second. The camera was positioned 4 m laterally from the walkway in a sagittal (lateral) view and was aligned to capture the entire 6-m measurement segment. This setup, which follows standard observational gait analysis techniques [25], enabled accurate visual identification of foot contact at both the start and end of the measurement zone. The camera angle, zoom, and position, as well as the floor markers, were kept fixed and consistent across all participants to ensure uniformity and reduce variability in gait analysis, including for calculations of the LRI and optimum walking speed (OWS).

### Gait Analysis and Parameter Calculation

To ensure objectivity and reliability, all video recordings were independently analyzed by 2 experienced physiatrists who were blinded to the study conditions and not involved in the recording process. Initial foot contact was defined as the moment the heel of the leading foot touched the ground on or beyond the starting line of the 6-m segment. The first completed step was then counted, followed by subsequent steps until the final foot contact on or beyond the end line of the segment. All identifications of foot contacts and steps were based on visual inspection of individual video frames, using heel strike as the reference point, in accordance with standard gait analysis protocols [25].

Gait parameters were calculated as follows: average gait speed (m/s) was calculated by dividing the 6-m distance by the time difference between the first foot contact and the last completed footstep, average cadence (steps per minute) was calculated as the number of completed steps divided by the total walking time (from the first foot contact to the last completed step), and average stride length (m) was calculated as average gait speed (m/s)×2×60/cadence (steps per minute) [25].

To ensure proper use of the device, all participants were given a standardized 5-minute familiarization period. During this time, they adjusted the distance of the laser line projection in front of their foot according to their personal preference, such as stride length or visual comfort. Participants were considered ready to begin the intervention once they reported feeling comfortable with the laser projection and could consistently step to the indicated distance. The same 5-minute period and readiness criteria were applied to all participants to ensure consistency across the intervention. Then, each patient would sit still for 10 minutes before starting the experiments to wash out the movement effect. Before beginning each trial, each participant took 10 alternating steps with both feet to warm up. One of the examiners followed the participants to prevent falls during all the tests.

### Outcome Measures

Efficacy was primarily measured by the improvement in gait speed between the laser-off and laser-on conditions. Secondary measures included changes in stride length, TUG test results, and the LRI. The LRI is a method to determine the relationship between self-selected gait speed and the OWS, which is the speed that uses the lowest metabolic energy expenditure [26]. The OWS (m/s) was calculated from √(0.25×9.81×0.54 of height). The LRI (in percentages) was equal to 100× walking speed/OWS. We chose gait speed as the primary outcome because it is a reliable and sensitive tool to represent individual mobility and functional status and is easy to follow up clinically [13].

### Statistical Analysis

Statistical analysis was performed using STATA (version 17; StataCorp LLC). A mixed linear regression model with adjusting the sequence effect was used to analyze the differences in gait speed, stride length, the TUG test, and the LRI between 2 conditions: walking with laser-off and walking with laser-on. Statistical significance was set at *P*<.05. Effect sizes were calculated using Hedges *g* to correct for small sample size bias, with values of 0.20, 0.50, 0.80, and 1.20 indicating small, moderate, large, and very large effects, respectively.

### Sample Size and Analysis

The sample size was calculated based on the improvement of gait speed from comparable studies [27], with α=.05 and β=0.8, equal to 10 patients. Therefore, we recruited and randomized 10 participants to see the feasibility and efficacy of this device.

This trial was conducted and reported in accordance with the CONSORT (Consolidated Standards of Reporting Trials) 2010 guidelines (Multimedia Appendix 1).

## Results

### Participant Flow

**Figure 2:**
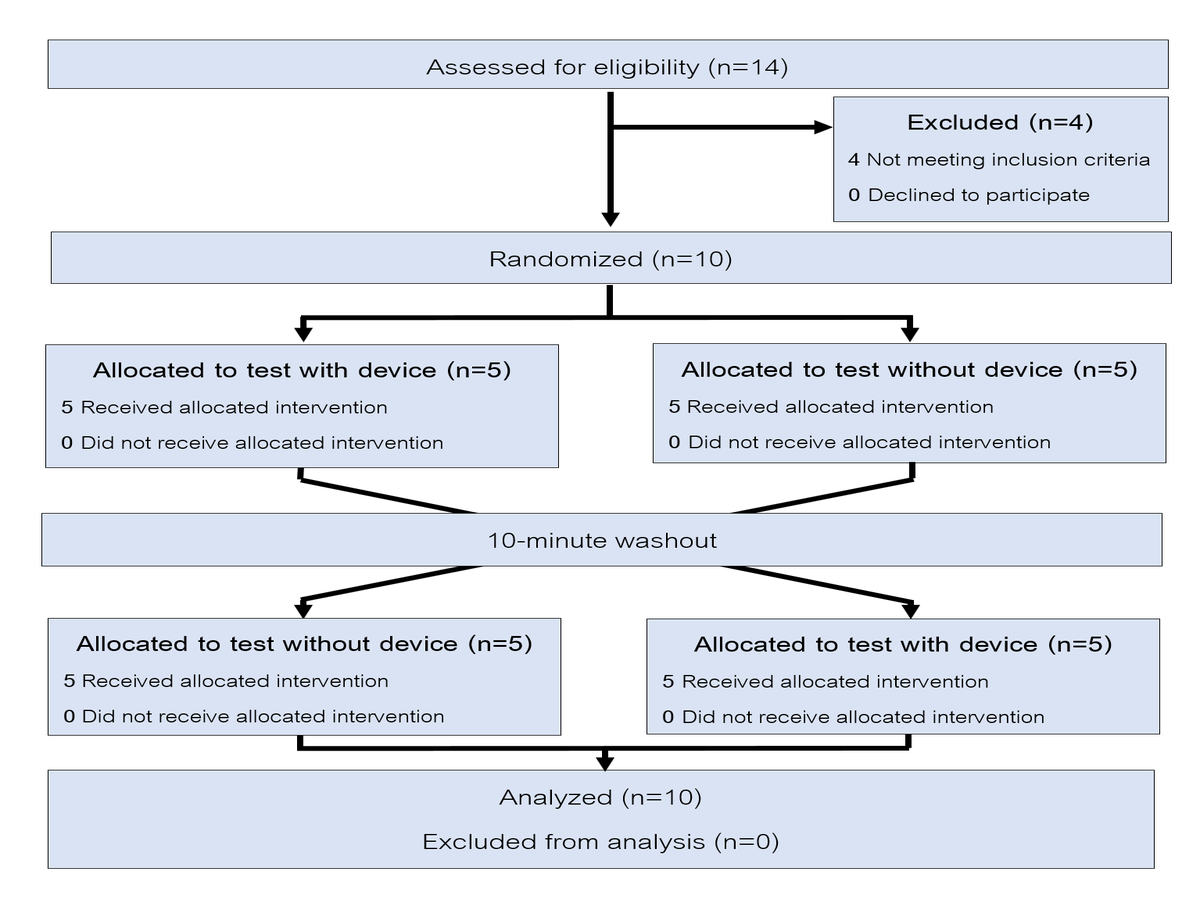
Flow diagram for crossover trials.

The flow diagram of this trial is shown in Figure 2.

### Participant Characteristics

In total, 10 of the 14 screened patients with parkinsonism who met the inclusion criteria were included in the study. Among these participants, 6 (60%) were diagnosed with PD, while the remaining 4 (40%) had nonprimary parkinsonism—specifically, 2 (20%) with secondary parkinsonism and 2 (20%) with atypical parkinsonism.

In total, 6 (60%) participants were able to walk without the use of a gait aid. A total of 9 of the 10 participants had not previously used any cueing devices. In total, 1 (10%) participant with vascular parkinsonism had created her own visual cue by attaching a piece of wood to her cane; however, she was unable to concentrate and use the device effectively. The participants’ clinical characteristics are summarized in Table 1.

**Table 1:** Demographic data of participants.

Data			Values
**Age (years)**			
	Mean (SD)		69 (7.2)
**Disease duration (years)**			
	Median (IQR)		5 (2.8-12.3)
**Sex, n (%)**			
	Male		5 (50)
	Female		5 (50)
**Hoehn and Yahr, n (%)**			
	2		2 (20)
	3		5 (50)
	4		3 (30)
**Parkinsonism (n=10), n (%)**			
	Parkinson disease		6 (60)
	**Atypical parkinsonism**		
		Progressive supranuclear palsy	1 (10)
		MSA-C^a^	1 (10)
	**Secondary parkinsonism**		
		Vascular parkinsonism	1 (10)
		Normal pressure hydrocephalus	1 (10)
**BMI (kg/m^**2**^)**			
	Mean (SD)		24.42 (5.9)
**Gait aid, n (%)**			
	Tripod cane		3 (30)
	Walker		1 (10)
	None		6 (60)

^a^MSA-C: multiple system atrophy cerebellar type.

### Gait Measures

Before comparing the results, the mixed linear regression model was used to test for a sequence effect—AB (laser-on followed by laser-off) versus BA (laser-off followed by laser-on)—for each parameter. Across all outcomes (gait speed, stride length, TUG, and LRI), no significant sequence effects were observed in patients with parkinsonism or within the PD and atypical parkinsonism subgroups, indicating that the order of laser-on and laser-off interventions had no impact on the results (Table S1 in Multimedia Appendix 2).

The results with the ankle bracelet laser showed statistically significant improvements in all parameters, with clinically meaningful differences in gait speed, stride length, and TUG test results. With the laser-on, gait speed and stride length improved by a mean of 0.07 m/s (95% CI 0.04-0.09 m/s; *P*<.001) and 0.17 m (95% CI 0.11-0.23 m; *P*<.001), respectively. The TUG test time decreased by a mean of 7.69 seconds (95% CI 2.82-12.55 seconds; *P*=.002). The LRI improved by a mean of 4.46% (95% CI 2.56%-6.36%; *P*<.001). The mean cadence with the laser-off was 98.55 (95% CI 81.32-115.78) steps per minute, whereas with the laser-on, it was 88.80 (95% CI 74.13-103.47) steps per minute. The overall results of the study are presented in Table 2.

**Table 2:** The outcomes comparison between laser-off and laser-on in 10 patients with parkinsonism.

Outcomes	Laser-off, mean (95% CI)	Laser-on, mean (95% CI)	Mean difference (95% CI)	*P* value
Gait speed (m/s)	0.60 (0.48-0.72)	0.66 (0.54-0.78)	0.07 (0.04-0.09)	<.001
Stride length (m)	0.73 (0.61-0.86)	0.90 (0.78-1.03)	0.17 (0.11-0.23)	<.001
TUG^a^ (seconds)	34.76 (19.68-49.84)	27.08 (12.00-42.16)	7.69 (2.82-12.55)	.002
LRI^b^ (%)	40.75 (32.57-48.93)	45.21 (37.02-53.39)	4.46 (2.56-6.36)	<.001

^a^TUG: timed up and go.

^b^LRI: locomotor rehabilitation index.

### Subgroup and Effect Size Analysis

After separating the participants into Parkinson and atypical Parkinson groups, improvements in all parameters were also observed, as detailed in Tables 3 and 4. Furthermore, Table 5, which shows the coefficient and effect size (Hedges *g*) for all parameters, indicates a large effect size for stride length (Hedges *g*=0.81). The other parameters showed small to medium effects.

**Table 3:** The outcomes comparison between laser-off and laser-on in 6 patients with typical Parkinson disease.

Outcomes	Laser-off, mean (95% CI)	Laser-on, mean (95% CI)	Mean difference (95% CI)	*P* value
Gait speed (m/s)	0.62 (0.43-0.82)	0.68 (0.49-0.88)	0.06 (0.01-0.10)	.01
Stride length (m)	0.73 (0.54-0.91)	0.92 (0.74-1.10)	0.20 (0.13-0.26)	<.001
TUG^a^ (seconds)	40.85 (16.85-64.86)	32.31(8.30-56.31)	8.55 (1.8-15.30)	.01
LRI^b^ (%)	42.53 (29.33-55.72)	46.43 (33.23-59.62)	3.89 (0.85-6.94)	.01

^a^TUG: timed up and go.

^b^LRI: locomotor rehabilitation index.

**Table 4:** The outcomes comparison between laser-off and laser-on in 4 patients with atypical Parkinson disease.

Outcomes	Laser-off, mean (95% CI)	Laser-on, mean (95% CI)	Mean difference (95% CI)	*P* value
Gait speed (m/s)	0.55 (0.48 to 0.62)	0.63 (0.57 to 0.70)	0.08 (0.07 to 0.09)	<.001
Stride length (m)	0.74 (0.67 to 0.83)	0.88 (0.80 to 0.96)	0.13 (0.03 to 0.23)	.01
TUG^a^ (seconds)	25.62 (20.11 to 31.14)	19.23 (13.71 to 24.75)	6.40 (–0.16 to 12.96)	.050
LRI^b^ (%)	38.07 (33.26 to 42.88)	43.38 (38.56 to 48.19)	5.30 (4.60 to 6.01)	<.001

^a^TUG: timed up and go.

^b^LRI: locomotor rehabilitation index.

**Table 5:** The coefficients and effect sizes (Hedges g) for all parameters.

Outcomes	Coefficient	Effect size, Hedges *g*
Gait speed (m/s)	0.07	0.33
Stride length (m)	0.17	0.81
TUG^a^ (seconds)	7.69	0.31
LRI^b^ (%)	4.46	0.33

^a^TUG: timed up and go.

^b^LRI: locomotor rehabilitation index.

### Subjective Aspects

There were no adverse effects while using the device, such as dizziness while looking at the laser line or blurred vision.

## Discussion

### Principal Findings

We developed the ankle bracelet laser as a new closed-loop visual cueing device to improve gait performance in patients with parkinsonism and FOG. Our study revealed the improvement of gait speed and stride length and a decrease in TUG time in the laser-on condition compared to the laser-off condition. No participants experienced walking difficulties or adverse effects while using the device.

### Efficacy and Clinical Significance

The improvement of gait speed by an average of 0.07 (95% CI 0.04-0.09) m/s reached the minimal clinically important difference for minimal change (0.06 m/s) in persons with PD [28]. Furthermore, in patients with H&Y stage 3, gait speed was improved by an average of 0.1 m/s, reaching this same minimal clinically important difference with a large effect size (Hedges *g*=0.75; Table S2 in Multimedia Appendix 2).

Gait speed is a key indicator for independent living and life in the community and is related to objective measures of balance such as the Berg balance, TUG, and severity in H&Y [29]. Slow gait speed and short stride length are related to falling and fragility in people with PD [1]. Therefore, increasing gait speed is related to greater mobility, increased independence, and improved balance for patients with parkinsonism. Stride length with laser-on showed a clinically significant average improvement of 0.17 m [1]. Increasing the stride length [1,30] also improves walking performance and reduces shuffling gait in FOG, which might help reduce the risk of falls. This study also showed the improvement in gait speed and stride length but decreased cadence after laser-on. These results are related to the walking characteristics of people with PD, in which gait speed is more related to stride length than cadence [1].

Our study found a clear change in these spatiotemporal parameters. In contrast, previous studies produced controversial or negative results [12,14,15,31]. Most previous mobile cueing devices need manual control and require attention and cognitive function to use the devices effectively. These may be impractical to some patients, especially those with cognitive impairments, which are commonly found in parkinsonism. Our ankle bracelet laser could project cues that automatically follow each walking step. Therefore, the demands on executive function and attention are lower [10,11] and may be the reason for the results in our study. In addition, the movable laser line following tibial movement might increase sensory input and improve motor performance, especially by increasing stride length [32]. This device also acts as a proof of concept for ease of use by leading to an immediate improvement in inexperienced participants; a total of 9 of 10 had no experience with visual cues, and only 1 person had a history of unsuccessful use of a self-made cue attached to a walking stick.

In the TUG test, the mean difference of 8.55 seconds reached the minimal detectable change in PD (3.5 seconds) [33,34]. The TUG test is one of the tools used to evaluate the risk of falls with good reliability and validity [35]; however, there was less evidence regarding visual cues and the TUG test [36]. Some studies have focused on sit-to-stand ability [37,38] and did not represent the dynamic balance in turning, which is usually impaired in patients with parkinsonism and FOG.

Our results suggest that the reduction in TUG time reflects improved balance and overall function by addressing the crucial phases—sit-to-stand, walking, turning, and stand-to-sit. Because FOG often occurs during initiation, turning, or directional changes, a shorter TUG time, particularly in the turning component, may have a direct and clinically meaningful impact on FOG reduction, since turning impairments are a frequent trigger for FOG and a common cause of falls [10,39]. These findings are consistent with improvements seen in previous studies using NW training [20] and align with recent research identifying TUG time and turning velocity as early indicators of gait abnormalities in parkinsonism [40]. Together, our results support the use of TUG time, alongside gait speed, as a primary outcome in future FOG intervention studies and highlight the potential for smartwatch-based monitoring to capture real-world events outside the clinic [41].

The higher LRI represents the improvement of the metabolic economy [26]. The LRI in our study was also improved, similar to the NW training, and reflected that walking speed in laser-on is closer to OWS and has a lower metabolic cost. However, the degree of improvement in our study was less than the 6-week NW training. In our study, 80% of the participants were in H&Y stages 3-4 with FOG episodes. On the other hand, the people in NW training were in an average H&Y stage of 1.5, with only a few individuals (3/16) experiencing FOG. Therefore, disease stage may influence the degree of improvement observed. The ankle laser may be more appropriate for individuals with advanced parkinsonism, whereas NW training appears most beneficial for improving walking economy in those with mild PD [2].

Due to the small sample size in each stage, further study should be done with a larger sample size in different H&Y staging to prove this hypothesis. Further study should also include coordinating the exercise training with the device, adjusting to each stage to see the different effects.

This study found significant improvements in patients with H&Y stages 2-4, with patients with H&Y stage 3 showing marked improvement in gait speed, TUG test, and the LRI with a large effect size, shown in Table S2 in Multimedia Appendix 2.

Our sample size of 10 was determined a priori based on power calculations from a previous study (α=.05 and β=0.8), which was sufficient to detect statistically significant improvements in all primary and secondary outcomes of this study. While we acknowledge that larger trials are ultimately necessary to achieve greater statistical power, this study was designed as an exploratory trial to evaluate the efficacy and feasibility of a novel device. Because of the small number of patients with each H&Y stage, future studies should examine these groups separately to identify any different outcomes, including long-term effects, between each stage. Based on sample size calculations from this study, a total of 39 participants would be required for a future crossover trial comparing the effects of this device in moderate to severe parkinsonism (H&Y stages 3-4).

Our device led to improvement in all 4 patients with atypical and secondary parkinsonism, which is very interesting because these patients have smaller responses to anti-parkinsonism drugs than patients with primary parkinsonism [42,43], and there is less evidence on the use of cueing devices in these groups. We should conduct further studies with a larger sample size of patients with different types of parkinsonism.

### Pathophysiology and Cognitive Considerations

Currently, the pathogenesis of FOG is still unclear. The basal ganglia are a group of nuclei that controlled the movement, such as walking from initiation to walk and maintain the walking process, by providing the internal cue via the connection pathway with the supplementary motor area [9]. It has been proposed that FOG is caused by frontal lobe dysfunction or a lack of connectivity between frontal lobe and the basal ganglia [27].

Visual cues could activate the dorsolateral premotor control system by bypassing the brain network via the cerebello-parieto-premotor loops instead of the usual route via the hypoactive basal ganglia-supplementary motor area. The single-photon emission computed tomography study [44] also showed more enhancement of cerebral blood flow in the right lateral premotor cortex, which is mainly influenced by cerebellar input [44], and middle frontal gyrus, as well as reduced left dorsolateral prefrontal cortex activity in patients with PD and FOG while walking on treadmills with visual cues compared to control.

The frontostriatal pathways [45] that connect the shifting between cognitive and motor control are less efficient in freezers than in nonfreezers [11]; thus, individuals with parkinsonism who experience FOG often have reduced cognitive reserve [16] and deficits in sequence learning [18]. These underlying cognitive issues lead to difficulties with dual-tasking and limit their ability for motor relearning.

Our ankle bracelet laser focuses on ease of use and natural walking to minimize cognitive overload, allowing patients to focus on gait training. This study did not compare the device directly with other visual cueing systems. Future trials should evaluate its efficacy relative to other systems to identify potential differences or add on data values.

### The Closed-Loop Ankle Bracelet Laser System

Similar to the systems described in Ginis et al [11] and Muthukrishnan et al [46], our device is a closed-loop cueing system that uses wearable sensors to detect leg motion and automatically activates a laser cue via a microcontroller. Through this process, the external (visual) cue was projected based on real-time movement, which was considered the motor feedback. The combination of the feedback from the user’s movement and the feedforward of the sensory stimuli may enhance the neuroplasticity and motor relearning. Therefore, incorporating the ankle bracelet laser into gait training or exercise programs should be investigated to determine its potential role in sensory feedback and motor relearning.

### Study Limitations and Future Directions

This study found an immediate and significant improvement with less than 5 minutes of trials, which suggests the ease and practical use of the ankle bracelet laser system. However, we still lack a study of using the device in a home setting, which should be further done to prove this concept in daily living. Cognitive impairment is an important factor related not only to the efficacy of visual cues but also to the degree of FOG. Future studies should also investigate different degrees of cognitive impairment and evaluate correlations between gait improvement, the benefits of visual cues, and cognitive function.

The other limitation of using a visual cue is the need to look down at the laser line while walking, which the ankle bracelet laser projects continuously. This device could be programmed to project the laser cue on demand, providing feedback to the user only when FOG occurs. However, there is still controversy when comparing the efficacy of continuous cueing with on-demand cueing. Although previous studies have reported better results with continuous cueing, the patients also reported greater fatigue while using continuous cues than when using on-demand cues [38]. Because the long-term effects of the 2 methods remain unknown, future studies should compare results using the ankle bracelet laser with on-demand cues, such as determining the optimal time to project the laser line and comparing the results with continuous laser projection [47].

### Conclusions

The ankle bracelet laser cue produced immediate improvements in gait speed, stride length, and balance in patients with parkinsonism and FOG, suggesting that the device can acutely enhance gait performance. Further research is needed to determine whether these benefits are sustained and applicable to daily life activities.

## Appendix

Multimedia Appendix 1 CONSORT 2010 checklist.

Multimedia Appendix 2 Table S1. Sequence effects from the mixed linear regression model for all outcome parameters (gait speed, stride length, Timed Up and Go [TUG], and lateral rhythmicity index [LRI]) in patients with Parkinsonism, Parkinson’s disease, and atypical Parkinsonism. AB = Laser-On followed by Laser-Off; BA = Laser-Off followed by Laser-On. Table S2. Outcomes comparison between laser-off and laser-on conditions for 5 patients with H&Y stage 3 Parkinsonism. This table includes the mean, mean difference, *P* value, Hedge g effect size, and interpretation for each outcome parameter.

## Abbreviations

CONSORT: Consolidated Standards of Reporting Trials
FOG: freezing of gait
H&Y: Hoehn and Yahr
LRI: locomotor rehabilitation index
NW: Nordic walking
OWS: optimum walking speed
PD: Parkinson disease
TUG: timed up and go
