# Supplementary material for: Ankle Bracelet Laser as a Novel Portable Device to Improve Walking in Patients With Parkinsonism: Randomized Crossover Controlled Trial
Source: JMIR Rehabil Assist Technol. 2025 Oct 7;12:e70209. doi: 10.2196/70209 (PMC12541267; doi:10.2196/70209)
Supplement: Multimedia Appendix 2 [file rehab_v12i1e70209_app2.docx]

**Table S1.** Sequence effects from the mixed linear regression model for all outcome parameters (gait speed, stride length, Timed Up and Go [TUG], and lateral rhythmicity index [LRI]) in patients with Parkinsonism, Parkinson’s disease, and atypical Parkinsonism. AB = Laser-On followed by Laser-Off; BA = Laser-Off followed by Laser-On.

| Outcome | Group | BA vs AB Coef. (95% CI) | P-value |
| --- | --- | --- | --- |
| Gait speed | Parkinsonism | 0.09 (−0.14, 0.33) | 0.44 |
|  | Parkinson’s Disease | 0.09 (−0.32, 0.50) | 0.66 |
|  | Atypical Parkinsonism | 0.07 (−0.09, 0.22) | 0.42 |
| Stride length | Parkinsonism | −0.12 (−0.36, 0.12) | 0.34 |
|  | Parkinson’s Disease | −0.26 (−0.64, 0.12) | 0.18 |
|  | Atypical Parkinsonism | 0.06 (−0.07, 0.20) | 0.37 |
| TUG | Parkinsonism | 6.92 (−22.85, 36.68) | 0.46 |
|  | Parkinson’s Disease | −4.09 (−54.51, 46.33) | 0.87 |
|  | Atypical Parkinsonism | 3.09 (−7.16, 13.34) | 0.55 |
| LRI | Parkinsonism | 6.98 (−9.27, 23.23) | 0.40 |
|  | Parkinson’s Disease | 8.27 (−19.54, 36.08) | 0.56 |
|  | Atypical Parkinsonism | 3.56 (−7.52, 14.64) | 0.53 |

**Table S2.** Outcomes comparison between laser-off and laser-on conditions for 5 patients with H&Y stage 3 Parkinsonism. This table includes the mean, mean difference, P-value, Hedges’ *g* effect size, and interpretation for each outcome parameter

| Outcomes | Mean (95% CI) | | Mean difference (95% CI) | P value | Hedges’ *g* | Interpretation |
| --- | --- | --- | --- | --- | --- | --- |
|  | Laser-off | Laser-on |  |  |  |  |
| Gait speed (m/s) | 0.60 (0.49, 0.70) | 0.70 (0.59, 0.81) | 0.10(0.08, 0.13) | <.001 | 0.75 | Large |
| Stride length(m) | 0.80 (0.63, 0.96) | 0.92 (0.76, 1.09) | 0.13 (0.07, 0.19) | <.001 | 0.61 | Moderate–large |
| TUG^a^, sec | 25.17 (20.83, 29.52) | 19.04 (14.69, 23.38) | 6.14 (0.96, 11.31) | .02 | 1.12 | Very large |
| LRI^b^(%) | 40.26 (33.98, 46.54) | 47.06(40.77, 53.34) | 6.80 (5.15, 8.44) | <.001 | 0.86 | Large |
